# Single-nucleotide polymorphism profiling by multimodal-targeted next-generation sequencing in methotrexate-resistant and -sensitive human osteosarcoma cell lines

**DOI:** 10.3389/fphar.2023.1294873

**Published:** 2023-11-22

**Authors:** Chiara Casotti, Claudia Maria Hattinger, Maria Pia Patrizio, Silvia Luppi, Leonardo Fantoni, Michela Pasello, Katia Scotlandi, Toni Ibrahim, Massimo Serra

**Affiliations:** ^1^ Osteoncology, Bone and Soft Tissue Sarcomas and Innovative Therapies, IRCCS Istituto Ortopedico Rizzoli, Bologna, Italy; ^2^ Department of Experimental, Diagnostic and Specialty Medicine (DIMES), University of Bologna, Bologna, Italy; ^3^ Laboratory of Experimental Oncology, IRCCS Istituto Ortopedico Rizzoli, Bologna, Italy

**Keywords:** osteosarcoma, methotrexate resistance, next-generation sequencing, single-nucleotide polymorphism, pharmacogenomics

## Abstract

**Introduction:** Methotrexate (MTX) is one of the most important drugs included in the first-line protocols to treat high-grade osteosarcoma (HGOS). Although several polymorphisms have been reported to be associated with drug response or MTX-related toxicity in pharmacogenetic studies, their role in the development of MTX resistance in HGOS is still unclear.

**Methods:** Therefore, in this study, 22 single nucleotide polymorphisms (SNPs) in 4 genes of the folate metabolism, 7 MTX transporter genes, and 2 SNPs of the tumor protein p53 (*TP53*) gene were investigated using a custom multimodal-targeted next-generation sequencing (mmNGS) approach in 8 MTX-resistant and 12 MTX-sensitive human HGOS cell lines. The panel was validated by TaqMan genotyping assays.

**Results:** High instability of TP53 rs1642785 was observed in all U-2OS/MTX variants. Allele changes of the solute carrier family 19 member 1/replication factor C subunit 1 (SLC19A1, previously known as RFC1) and rs1051266 were identified in all Saos-2/MTX-resistant variants in both DNA- and RNA- derived libraries compared to the parental Saos-2 cell line. Allele changes of methylenetetrahydrofolate reductase (MTHFR) rs1801133 were identified only in the RNA-derived libraries of the two U2OS variants with the highest MTX resistance level. Significantly upregulated gene expression associated with the development of MTX resistance was revealed for dihydrofolate reductase (DHFR) whereas SLC19A1 was downregulated. In addition, a fusion transcript of DHFR (ex4) and MutS Homolog 3 (MSH3) (ex9) was identified in the RNA libraries derived from the two U-2OS variants with the highest MTX resistance level.

**Conclusion:** This innovative mmNGS approach enabled the simultaneous exploration of SNPs at DNA and RNA levels in human HGOS cell lines, providing evidence of the functional involvement of allele changes associated with the development of MTX resistance.

## 1 Introduction

High-grade osteosarcoma (HGOS) is a very aggressive malignant bone tumor arising in children and young adults. Conventional first-line treatment for HGOS is based on neoadjuvant chemotherapy with methotrexate, doxorubicin, and cisplatin, with the possible addition of ifosfamide (Gill and Gorlick, 2021). As clinical treatment response to conventional drugs is limited by the development of intrinsic or acquired drug resistance, it is of fundamental importance to understand which mechanisms induce unresponsiveness to these chemotherapeutic agents. Methotrexate (MTX) is one of the most widely used drugs for HGOS treatment. MTX is a folate analog that targets dihydrofolate reductase (DHFR), a member of the reductase enzyme family that takes part in the metabolism of purines and pyrimidines. MTX is commonly used at high dosages, which may often cause adverse treatment-related effects, such as nephrotoxicity, hepatotoxicity, mucositis, and bone marrow suppression (Howard et al., 2016). Revealing the factors involved in MTX resistance or MTX-related toxicities will help enhance cell sensitivity toward the drug by simultaneously decreasing the risk of adverse toxic effects.

Several studies have reported that the most relevant mechanisms for MTX resistance in HGOS are the decreased expression of the solute carrier family 19 member 1/replication factor C subunit 1 (SLC19A1, previously known as RFC1), which negatively affects the transportation of the drug inside the cell, and the increased expression of DHFR that limits the inhibitory efficacy of MTX (Guo et al., 1999; Hattinger et al., 2003; Hattinger et al., 2009).

Pharmacogenetic and pharmacogenomic studies in HGOS have identified gene polymorphisms that play a fundamental role in MTX metabolism and transport (Jabeen et al., 2015; Hegyi et al., 2017) and suggested that variations in these genes could be involved in the acquisition of MTX resistance.

Several studies reported methylenetetrahydrofolate reductase (MTHFR) rs1801133 as a polymorphism associated with the development of treatment-related adverse toxicity (Hattinger et al., 2017; Hurkmans et al., 2022). Furthermore, polymorphism rs1051266 of SLC19A1 was reported to be associated with clinical outcomes, reporting better survival and lower predisposition to develop metastasis for patients with the G allele (Windsor et al., 2012; Jabeen et al., 2015).

In this study, we have focused on single-nucleotide polymorphisms (SNPs) affecting genes involved in MTX transport, folate metabolism, MTX-related toxicity, and TP53 (Supplementary Table S1), and explored them in 8 MTX-resistant and 12 drug-sensitive human HGOS cell lines. The status of these polymorphisms was evaluated on DNA and RNA to investigate if the genetic variations were also maintained in the transcriptome. SNPs that changed their genotype in relation to drug resistance were expected to play an important role in the development of MTX unresponsiveness. This analysis was performed by an innovative multimodal-targeted next-generation sequencing (mmNGS) approach that allowed for simultaneously revealing the presence of SNPs in both DNA and RNA and also providing information on the gene expression level and the possible presence of fusion transcripts.

## 2 Materials and methods

### 2.1 Cell lines

The U-2OS and Saos-2, MG-63, and HOS human HGOS cell lines were obtained from the American Type Culture Collection (ATCC, Rockville, MD, United States). The IOR/OS9, IOR/OS10, IOR/OS14, IOR/OS15, IOR/OS18, IOR/OS20, IOR/MOS, and IOR/SARG human HGOS cell lines were established and characterized, as previously described (Benini et al., 1999), at the Laboratory of Experimental Oncology of the Orthopedic Rizzoli Institute (Bologna, Italy).

The variants resistant to MTX were established by exposing the sensitive cell lines, such as U-2OS and Saos-2, to *in vitro* step-by-step increasing MTX concentrations. The *in vitro* continuous drug exposure resulted in the establishment of variants which are resistant to 3 ng/mL MTX (U-2OS/MTX3), 30 ng/mL MTX (U-2OS/MTX30 and Saos-2/MTX30), 100 ng/mL MTX (U-2OS/MTX100 and Saos-2/MTX100), 300 ng/mL MTX (U-2OS/MTX300 and Saos-2/MTX300), and 1 μg/mL MTX (Saos-2/MTX1µg). These MTX concentrations corresponded to 0.01 (3 ng/mL), 0.07 (30 ng/mL), 0.22 (100 ng/ul), 0.66 (300 ng/ul), and 2.20 µM MTX (1 μg/mL) (Hattinger et al., 2003; Serra et al., 2004).

MTX-resistant variants have been cultured in Iscove’s modified Dulbecco’s medium (IMDM), supplemented with penicillin (100 U/mL), streptomycin (100 μg/mL), 10% fetal bovine serum (BioWhittaker Europe, Cambrex-Verviers, Belgium), and with an appropriate MTX concentration, and then maintained in a humidified atmosphere with 5% CO_2_ at 37°C. All the cell lines were found to be mycoplasma-free using a MycoAlert *Mycoplasma* Detection Kit (Lonza Italia, Milan, Italy).

DNA fingerprint analyses were performed for all cell lines using 17 polymorphic short tandem repeat sequences, confirming their identity (PowerPlex ESX 17 Fast System, Promega Italia, Milan, Italy).

### 2.2 Extraction of nucleic acids

Both DNA and RNA were isolated from frozen cell pellets. The extraction of nucleic acids was performed using the AllPrep DNA/RNA Mini Kit (QIAGEN, Hilden, Germany), according to the manufacturer’s instructions. After isolation, DNA and RNA concentrations were determined by spectrophotometry (NP-80, Implen GmbH, Munich, Germany), and the quality of all RNA samples was evaluated using the 2100 Bioanalyzer system (Agilent, Santa Clara, CA, United States) using the RNA 6000 kit (Agilent, Santa Clara, CA, United States).

### 2.3 Custom multimodal-targeted next-generation sequencing

Library preparation was performed for all 20 cell lines, according to the QIAseq Multimodal Panel handbook v06/2020 (QIAGEN, Hilden, Germany), as previously reported (Hattinger et al., 2022). The primers for DNA-derived libraries were designed for the analysis of 22 SNPs affecting 12 genes, which were related to MTX transport, folate metabolism, MTX-related toxicity, and TP53. The primers for RNA-derived libraries were designed for those SNPs mapping to coding regions and covering the entire 12 selected genes. This approach allowed not only the identification of DNA and RNA variations but also the assessment of the gene expression level and the detection of fusion transcripts.

For library preparation, the input amount was 40 ng of DNA and 100 ng of RNA. The libraries were quantified through the QIAseq Library Quant Assay kit (QIAGEN, Hilden, Germany) on a real-time PCR system (7900HT Fast Real-Time PCR system; Thermo Fisher Scientific by Life Technologies Italia, Monza, Italy). The libraries were then diluted to 1.2 p.m., pooled together, and sequenced on a NextSeq 500 system (Illumina Inc., San Diego, CA, United States) using a Mid-Output Reagent Kit v2.5 (300 cycles).

### 2.4 NGS data analysis by CLC genomics workbench

FastQ files were downloaded from the BaseSpace cloud (Illumina Inc., San Diego, CA, United States) and imported into the CLC genomics workbench (GWB) v22.0.1 (QIAGEN Bioinformatics, Aarhus, Denmark). Through the QIAseq Multimodal Analysis workflow, DNA variants, gene expression, and transcript fusions were identified. For the identification of RNA variants, a custom workflow was developed by the QIAGEN bioinformatics support.

**FIGURE 1:**
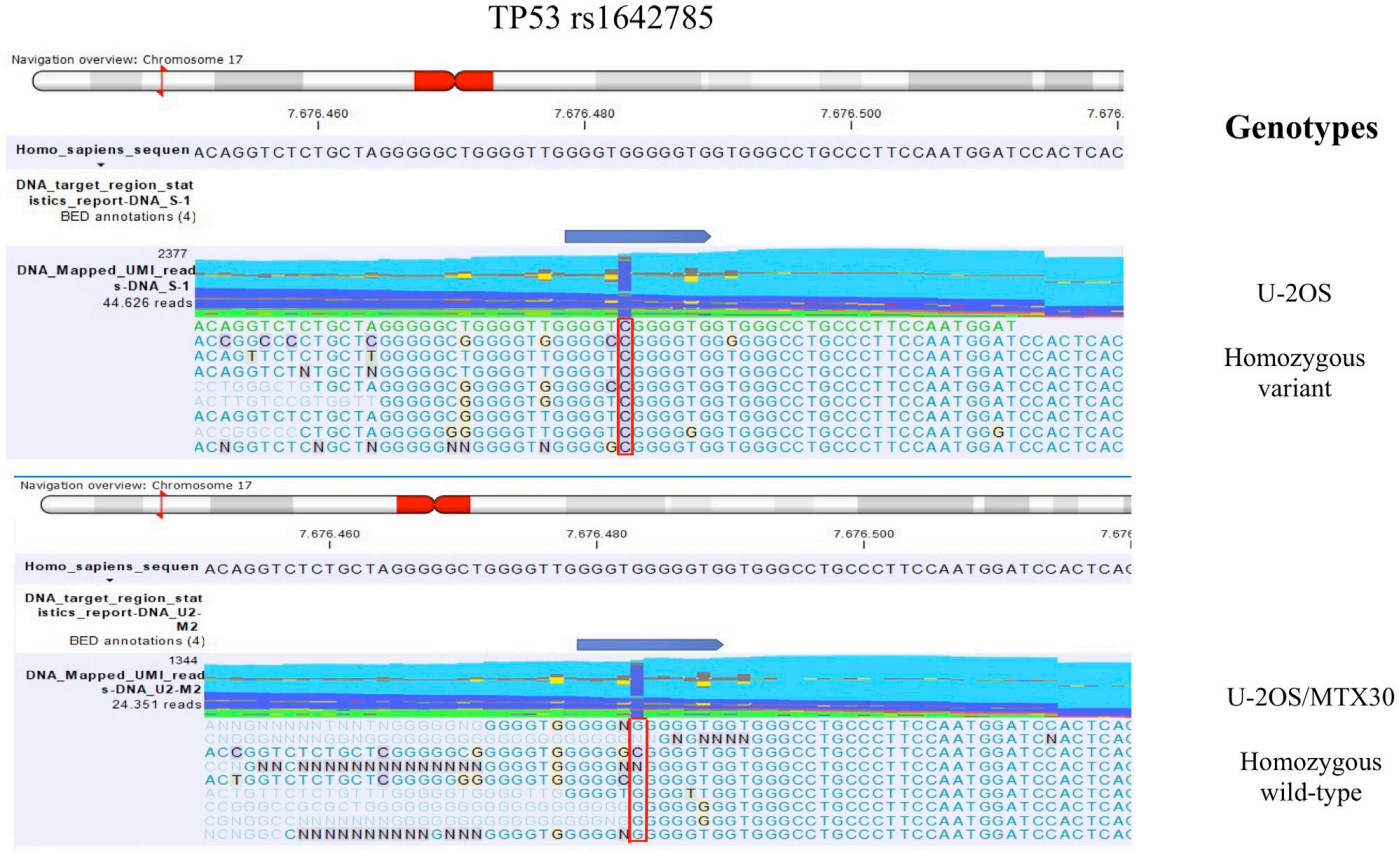
TP53 rs1642785 genotype identified by mmNGS in U-2OS and the MTX-resistant variant U-2OS/MTX30. The figure shows the genotype of TP53 rs1642785 that changed between the parental cell line U-2OS and its MTX-resistant variants. For example, the genotypes of parental U-2OS cells and the U-2OS/MTX30 variant are shown with a plot generated by CLC GWB software. The polymorphism status is highlighted with a red box.

**FIGURE 2:**
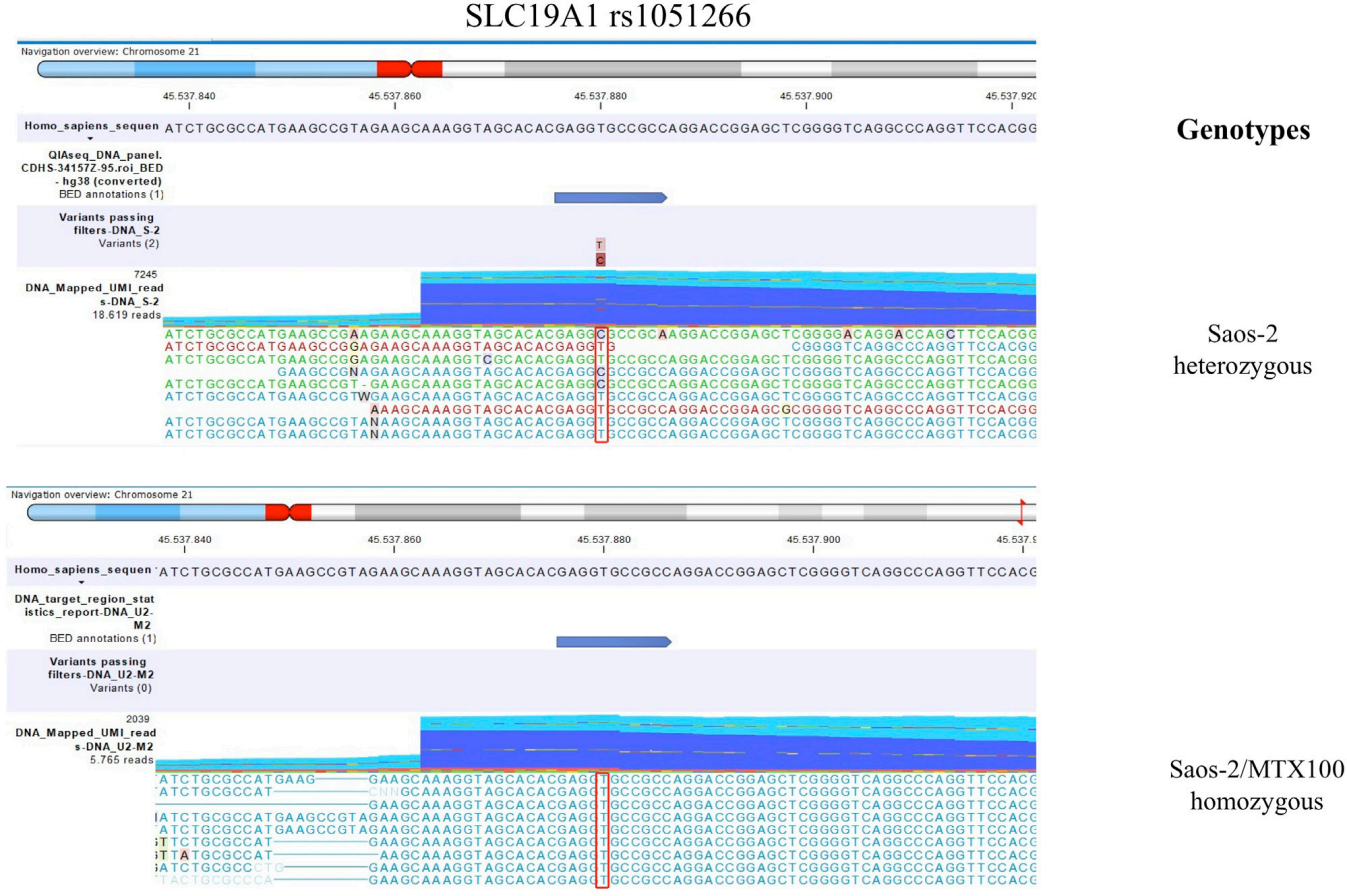
This figure shows the genotype of SLC19A1 rs1051266 that changed between the parental cell line Saos-2 and its resistant variant Saos-2/MTX300. The polymorphism is highlighted with a red box.

**FIGURE 3:**
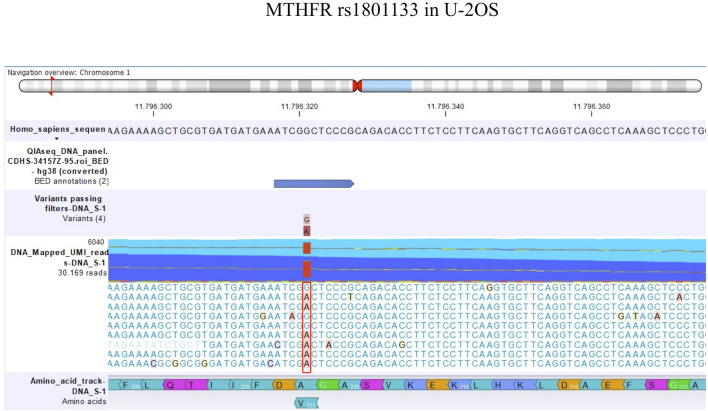
Amino acid change in MTHFR rs1801133 in U-2OS.

**FIGURE 4:**
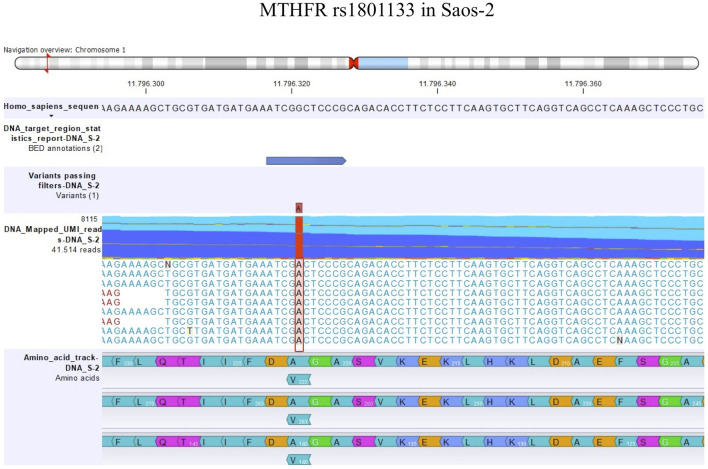
Amino acid change in MTHFR rs1801133 in Saos-2.

The detection of DNA and RNA variants was performed by aligning the reads to the human genome hg38 as a reference sequence and filtering using coverage of 100x and a variant allele frequency (VAF) higher than 3%. Figures 1, 2; Figures 3, 4 show the reads aligned to the reference genome hg38, where the polymorphisms have been highlighted with a red box. Paired reads are colored in blue, whereas mismatches between the reads and the reference are colored, following the RasMol structure color scheme.

For the analysis of differential gene expression, the transcripts per million data were considered, and a log_2_fold-change between the drug-resistant variants and their respective parental cell lines was evaluated using the differential expression tools of CLC GWB. A Bonferroni correction was used, and a *p*-value <0.05 was considered significant.

The hierarchical cluster tool of CLC GWB was used to create the heatmap setting, with the parameters Euclidean distance and complete linkage.

The principal component analysis (PCA) was conducted using PCA for the RNA-seq tool of CLC GWB, considering the 12 genes for all 20 cell lines to identify the clustering of the cell lines.

### 2.5 Single-nucleotide polymorphism genotyping by real-time PCR

To validate the sequencing data, a genotyping analysis was undertaken by TaqMan SNP genotyping and drug-metabolizing enzyme (DME) assays (Thermo Fisher Scientific by Life Technologies Italia, Monza, Italy), which are listed in Supplementary Table S2. This analysis was performed for 19 out of 22 selected polymorphisms by using 10 ng DNA as the input material. The genotyping experiments were performed on the VIIA 7 DX real-time PCR system (Thermo Fisher Scientific by Life Technologies Italia, Monza, Italy), and the results were analyzed with TaqMan Genotyper software (Thermo Fisher Scientific by Life Technologies Italia, Monza, Italy).

## 3 Results

### 3.1 Validation of mmNGS variant calling by genotyping

For each polymorphism, the data obtained by the mmNGS approach were compared with the data obtained by TaqMan genotyping. Supplementary Table S3 shows, for each cell line, this comparison considering only the allele with a VAF >3%. In the aforementioned Table, data on those polymorphisms that were identified as heterozygous or homozygous variants were reported.

From the comparison between the genotyping data and the sequencing results, we found that for 13/20 (65%) cell lines, the match was 100%, whereas for 7/20 (35%) cell lines the match was 95%. Overall, we found a high concordance between the data obtained by genotyping and sequencing approaches, and therefore, the performance of our custom panel for DNA variant calling can be considered reliable.

### 3.2 SNPs that change their genotype status in relation to MTX resistance

#### 3.2.1 Comparison between U-2OS MTX-resistant variants and parental cell line at the DNA level

The comparison of DNA variant calling data between U-2OS and its MTX-resistant variants identified a change in the polymorphism status of TP53 rs1642785. In the parental cell line, the genotype of TP53 rs1642785 was a homozygous variant, which changed during the acquisition of MTX resistance (Table 1).

**TABLE 1:** TP53 DNA single-nucleotide polymorphism that changed the genotype in U-2OS methotrexate-resistant variants compared to the parental cell line.

Cell line	TP53 rs1642785
U-2OS	CC
U-2OS/MTX3	CG
U-2OS/MTX30	GG
U-2OS/MTX100	GG
U-2OS/MTX300	CG

In addition, five sites of SNPs of the *TP53* gene were revealed to be near to the rs1642785 locus (Chr17: 7676471-7676,491), with a VAF higher than 3% (Figure 1). This observation is in accordance with the frequent TP53 variations found in the germline DNA of HGOS patients (Mirabello et al., 2015).

#### 3.2.2 Comparison between Saos-2 MTX-resistant variants and the parental cell line at the DNA level

By comparing the group of Saos-2 resistant variants to their parental cell line, a change of the SLC19A1 rs1051266 polymorphism was observed (Table 2).

**TABLE 2:** SLC19A1 DNA single-nucleotide polymorphism that changed the genotype in Saos-2 methotrexate-resistant variants compared to the parental cell line.

Cell line	SLC19A1 rs1051266
Saos-2	CT
Saos-2/MTX30	TT
Saos-2/MTX100	TT
Saos-2/MTX300	TT
Saos-2/MTX1µg	TT

In the sensitive cell line Saos-2, the genotype of SLC19A1 rs1051266 has proven to be a heterozygous variant (CT), while all four MTX-resistant variants were homozygous wild types (TT). Furthermore, Figure 2 shows this SNP in Saos-2 and Saos-2/MTX300 variants.

These findings suggest that this transition emerged during the first phases of MTX resistance acquisition.

### 3.3 Comparison of variant calling on DNA and RNA

#### 3.3.1 Variant calling in U-2OS MTX-resistant variants and U-2OS cell lines

The polymorphism status revealed on DNA was identified to be equal at the RNA level in 96% of the polymorphisms, indicating that these variants were also maintained during transcription. Table 3 shows the three SNPs presenting at least one variant allele that were identified in the MTX-resistant and sensitive cell lines both on DNA and RNA but did not change during the acquisition of MTX resistance.

**TABLE 3:** Polymorphisms that have at least one variant allele and remain stable in the sensitive and methotrexate-resistant U-2OS cell lines.

Cell line	Identified variants		
	ABCB1 rs2032582 DNA/RNA	MTHFR rs1801131 DNA/RNA	TP53 rs1042522 DNA/RNA
U-2OS	CA/CA	GT/GT	CC/CC
U-2OS/MTX3	CA/CA	GT/GT	CC/CC
U-2OS/MTX30	CA/CA	GT/GT	CC/CC
U-2OS/MTX100	CA/CA	GT/GT	CC/CC
U-2OS/MTX300	CA/CA	GT/GT	CC/CC
**Amino acid change due to a variant**			
U-2OS	Ser-Ala	Glu-Ala	Pro-Arg
U-2OS/MTX3	Ser-Ala	Glu-Ala	Pro-Arg
U-2OS/MTX30	Ser-Ala	Glu-Ala	Pro-Arg
U-2OS/MTX100	Ser-Ala	Glu-Ala	Pro-Arg
U-2OS/MTX300	Ser-Ala	Glu-Ala	Pro-Arg

ABCB1 rs2032582 was found to be a heterozygous variant with a consequent amino acid change of a Ser-Ala in both parental and resistant cell lines (Table 3). This gene plays a key role in the MTX level inside normal cells, being present in the liver cells where it pumps MTX out of the cell.

Another polymorphism that was found to be a heterozygous variant in all cell lines was MTHFR r1801131, which caused a Glu to Ala amino acid change (Table 3). This variation was reported to be associated with a decreased enzyme activity (Lopez-Lopez et al., 2013).

TP53 rs1042522 was found to be a homozygous variant in all cell lines, suggesting a possible biological advantage for cells carrying this allele. This SNP has been reported as a somatic mutation in HGOS, where it has been described to be associated with poor survival, supporting a more aggressive behavior of tumors carrying the variant allele (Pakos et al., 2004). By contrast, for MTHFR rs1801133, the status differed between DNA and RNA (Table 4). At the DNA level, the genotype remained a heterozygous variant (AG) in all cell lines, whereas at the RNA level, it was a heterozygous variant (AG) in the sensitive cell line and the two variants with lower MTX resistance but changed to a homozygous wild-type in the two more resistant variants. This SNP caused an amino acid change from Ala to Val that was lost in U-20S/MTX100 and U-2OS/MTX300 variants (Figure 3).

**TABLE 4:** Polymorphism MTHFR rs1801133 in U-2OS methotrexate-resistant variants and their parental cell line that changed at the RNA level. Polymorphism changes at the RNA level associated with MTX resistance have been highlighted in bold.

Cell line	MTHFR rs1801133 DNA/RNA
U-2OS	AG/AG
U-2OS/MTX3	AG/AG
U-2OS/MTX30	AG/AG
U-2OS/MTX100	AG/**GG**
U-2OS/MTX300	AG/**GG**
**Amino acid change due to a variant**	
U-2OS	Ala-Val
U-2OS/MTX3	Ala-Val
U-2OS/MTX30	Ala-Val
U-2OS/MTX100	Ala (no change)
U-2OS/MTX300	Ala (no change)

#### 3.3.2 Variant calling in Saos-2 MTX-resistant variants and Saos-2 cell lines

In Saos-2 cell lines, we observed that the genotype status occurring on DNA was maintained in 94.7% at the RNA level. Most of the polymorphisms were homozygous wild type with the exception of ABCB1 and MTHFD1, which showed a heterozygous and homozygous genotype, respectively. These variations were also identified at the RNA level (Table 5).

**TABLE 5:** Polymorphisms that remain stably mutated in the sensitive and methotrexate-resistant Saos-2 cell lines.

Cell line	Identified variants	
	ABCB1 rs2032582 DNA/RNA	MTHFD1 rs2236225 DNA/RNA
Saos-2	CA/CA	AA/AA
Saos-2/MTX30	CA/CA	AA/AA
Saos-2/MTX100	CA/CA	AA/AA
Saos-2/MTX300	CA/CA	AA/AA
Saos-2/MTX1µg	CA/CA	AA/AA
**Amino acid change due to a variant**		
Saos-2	Ser-Ala	Arg-Gln
Saos-2/MTX30	Ser-Ala	Arg-Gln
Saos-2/MTX100	Ser-Ala	Arg-Gln
Saos-2/MTX300	Ser-Ala	Arg-Gln
Saos-2/MTX1µg	Ser-Ala	Arg-Gln

By contrast, the status of one SNP, namely, MTHFR rs1801133, changed between DNA and RNA (Table 6). The MTHFR rs1801133 status was a homozygous variant (AA) in both sensitive and MTX-resistant cell lines on the DNA level with the consequent substitution of Ala with Val (Figure 4), whereas at the RNA level, this polymorphism emerged to be a heterozygous variant in all cell lines. Whether this status change, which was revealed only on the RNA level, regardless of MTX resistance, has a biological consequence or is due to the genetic heterogeneity and thresholds used for variant identification remains to be clarified by further studies.

**TABLE 6:** Polymorphism MTHFR rs1801133 in Saos-2 methotrexate-resistant variants and their parental cell line that changed at the RNA level.

Cell line	MTHFR rs1801133 DNA/RNA
Saos-2	AA/AG
Saos-2/MTX30	AA/AG
Saos-2/MTX100	AA/AG
Saos-2/MTX300	AA/AG
Saos-2/MTX1µg	AA/AG
**Amino acid change due to a variant**	
Saos-2	Ala-Val
Saos-2/MTX30	Ala-Val
Saos-2/MTX100	Ala-Val
Saos-2/MTX300	Ala-Val
Saos-2/MTX1µg	Ala-Val

### 3.4 Differential gene expression analysis assessed by targeted RNA-seq analysis

**FIGURE 5:**
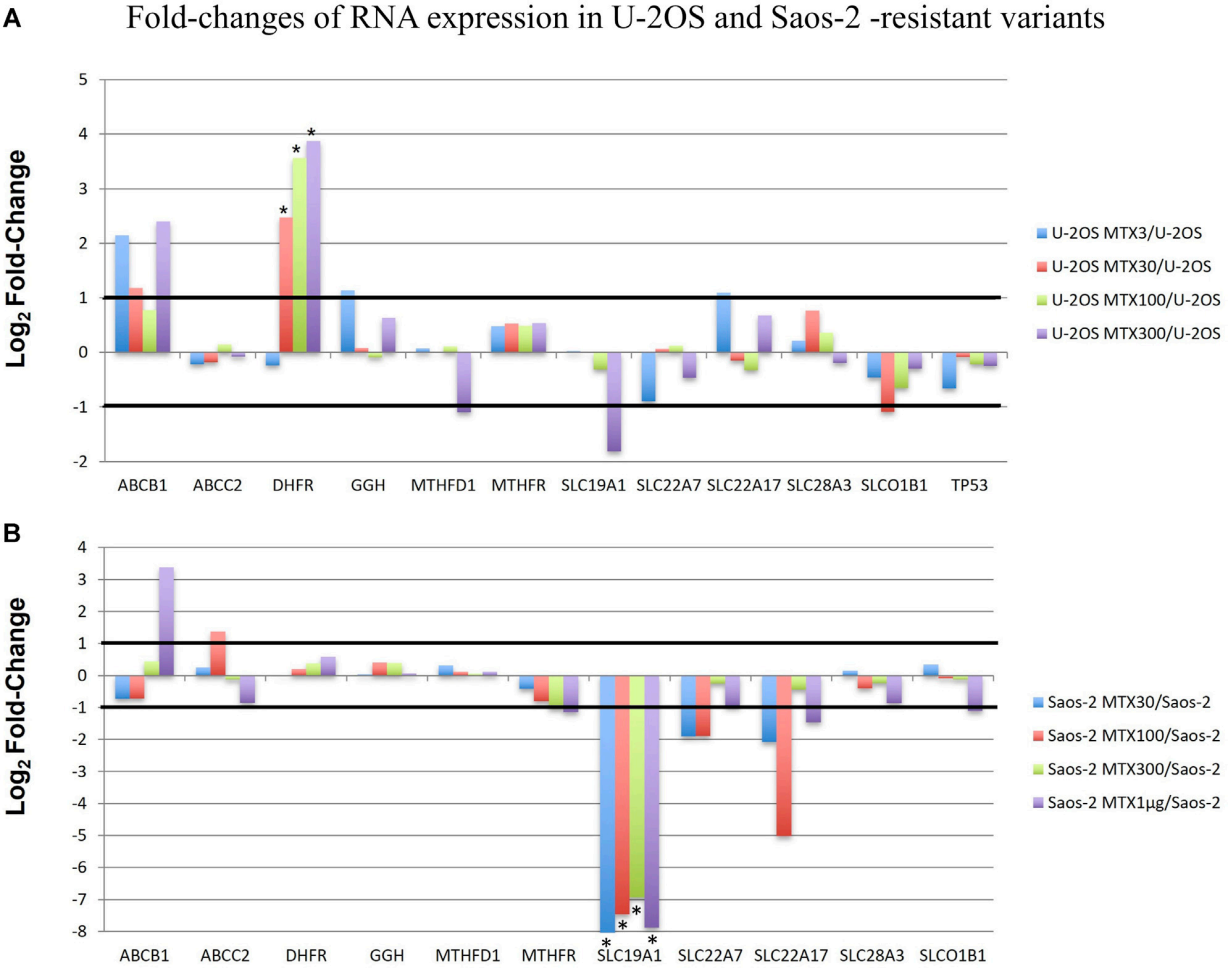
Fold changes of RNA expression in U-2OS/MTX-resistant variants **(A)** and Saos-2 MTX-resistant variants **(B)** compared with their parental cell lines. Thresholds for overexpression and underexpression are indicated by lines. Since the fold change values are based on a log2 scale, 1 and −1 values correspond to 2-fold increase or decrease, respectively. Significant changes are indicated by a *.

RNA-derived libraries were synthesized with primers that are specific for the aforementioned 12 genes and were analyzed with the RNA expression tool of CLC GWB. Figure 5 shows the fold changes in the gene transcription level in each MTX-resistant variant compared to its parental cell line.

In U-2OS/MTX-resistant variants, DHFR was significantly upregulated compared to U-2OS in parallel with the increasing MTX resistance. In addition, ABCB1 was also upregulated but did not reach significance.

On the contrary, in Saos-2 resistant variants, SLC19A1 was significantly downregulated in all MTX-resistant variants, with a *p*-value <0.05 after Bonferroni correction.

**FIGURE 6:**
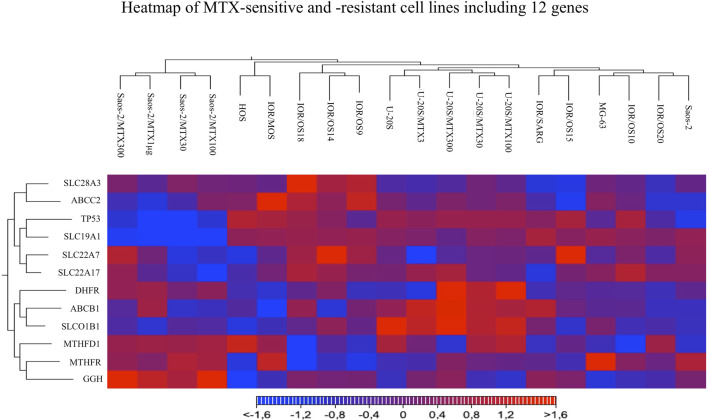
Heatmap analysis of all MTX-sensitive and -resistant human HGOS cell lines, including 12 genes, using the heatmap tool of the CLC genomics workbench.

Heatmap and hierarchical clustering analysis including all the studied cell lines and considering the expression level of all 12 genes were performed. The resulting clusterization showed that 20 cell lines can be divided into four main groups based on different gene expression levels (Figure 6).

Two groups included the sensitive cell lines, whereas the other two included the MTX-resistant variants. The U-2OS parental cell line and its relative resistant variants were grouped and characterized by the elevated expression of DHFR, ABCB1, and SLCO1B1. These data were in agreement with the fold changes shown in Figure 5. The cluster formed by U-2OS and its four MTX-resistant variants differed from the other cell lines due to the high expression of DHFR in U-2OS/MTX100 and U-2OS/MTX300, the expression of ABCB1, which was mainly overexpressed in U-2OS/MTX3 and U-2OS/MTX300, and due to SLCO1B1, which was overexpressed in U-2OS/MTX100 and U-2OS/MTX300. Figure 5 shows that the latter did not emerge as overexpressed in MTX-resistant variants because the SLCO1B1 expression level was higher in the parental U-2OS cell line.

Figure 5 shows that all four Saos-2 MTX-resistant variants were characterized by an underexpression of SLC19A1. However, differently from U-2OS, Saos-2 variants were grouped together but distant from their parental Saos-2 cell line. This long distance was due to different expressions of gamma-glutamyl hydrolase (GGH), which was highly overexpressed in all Saos-2 MTX-resistant variants.

**FIGURE 7:**
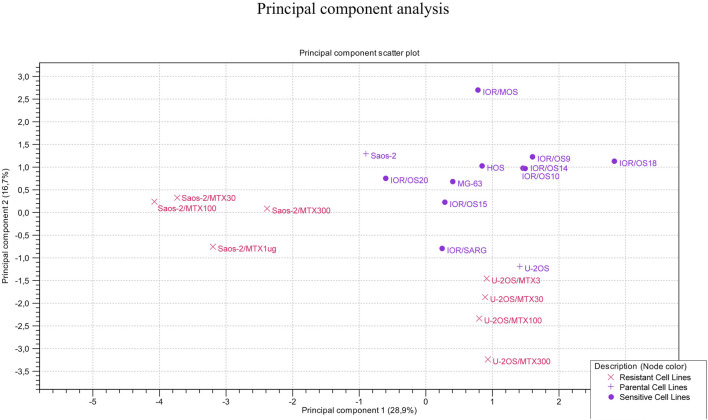
Principal component analysis on the 20 MTX-resistant and -sensitive cell lines.

The clustering of the two groups of MTX-resistant variants similar to their parental MTX-sensitive cell lines was also observed by PCA (Figure 7). Sensitive cell lines were positioned in the same region of the graph but not as a tight cluster, reflecting the typical heterogeneity of osteosarcoma.

### 3.5 Detection of fusion transcripts

**FIGURE 8:**
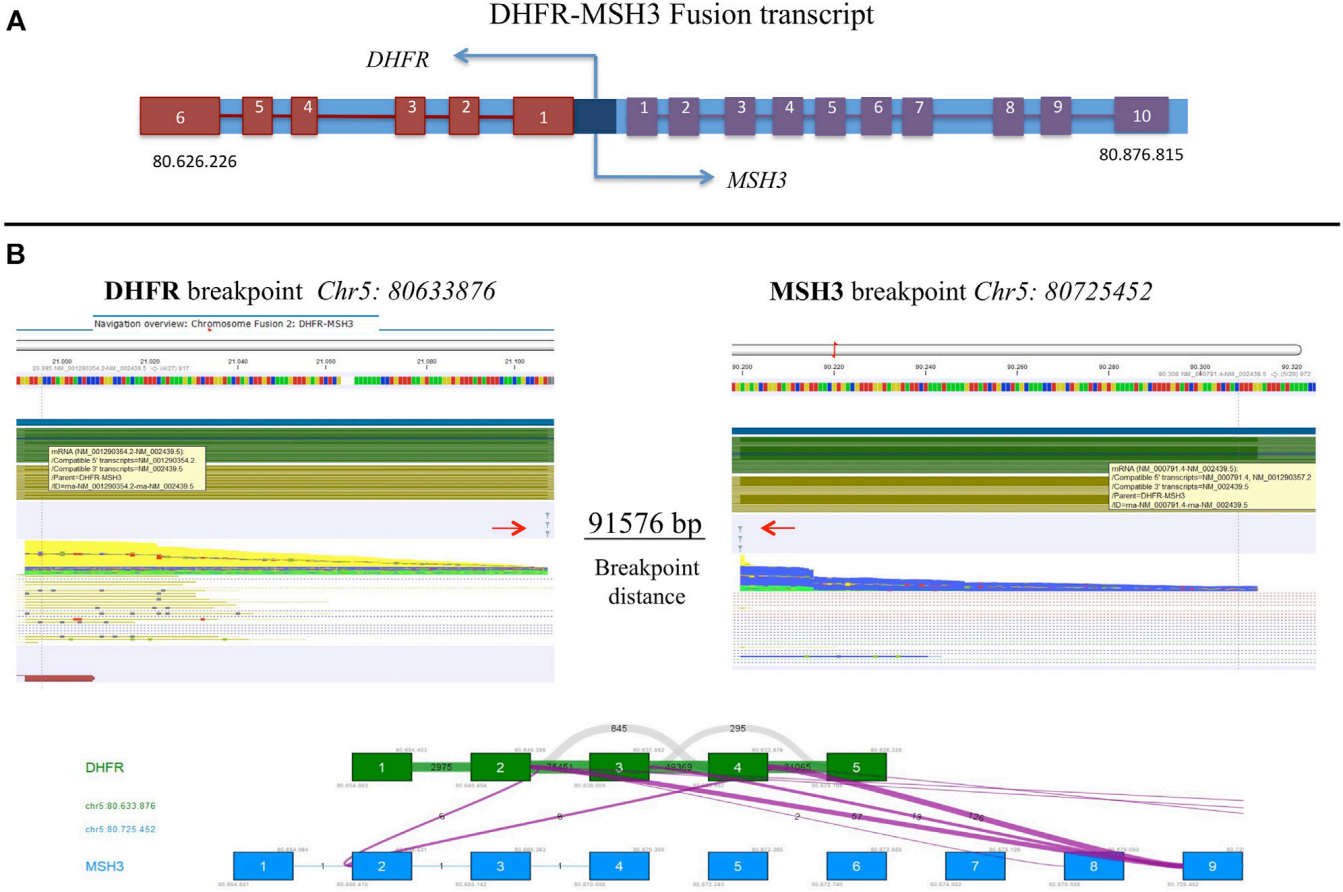
**(A)** Schematic representation of DHFR and MSH3 organization reconstructed according to Ensembl, indicating the position on chromosome 5. **(B)** Representation of the intrachromosomal fusion transcript between DHFR and MSH3. Arrows indicate breakpoints.

FastQ files of all RNA-derived libraries were explored for the presence of fusion transcripts using the QIAseq Multimodal Analysis of CLC GWB. One fusion transcript involving DHFR and MSH3 genes was identified in the U-2OS/MTX300 variant, the variant with the highest MTX resistance level (Table 7). A graphic representation of the particular chromosomal organization of DHFR and MSH3 genes is shown in Figure 8A. These two genes are on the same chromosome and are located closely to each other. MSH3 and DHFR genes are divergently transcribed from a promoter region that is shared by both of them (Drummond et al., 1997).

**TABLE 7:** Report of the fusion transcript between DHFR and MSH3 in the U-2OS/MTX300 cell line.

Fusion name	DHFR-MSH3
5′ Gene	DHFR
5′ Chromosome	5
3′ Gene	MSH3
3′ Chromosome	5
Reported transcript 5′	NM_001290354.2
Reported transcript 3′	NM_002439.5
Translocation name	DHFR{NM_001290354.2}:r.1_929_MSH3{NM_002439.5}: r.1417_4443
*p*-value	0,00
Z-score	15,34
Fusion crossing reads	126
5′ Read coverage	31.201
3′ Read coverage	196

5′ position	3′ position	Translocation name	Count
80.633.876	80.725.452	DHFR{NM_001290354.2}:r. 1_929_MSH3{NM_002439.	126
		5}:r.1417_4443	
80.649.388	80.725.452	DHFR{NM_001290354.2}:r. 1_686_MSH3{NM_002439.	57
		5}:r.1417_4443	
80.637.882	80.725.452	DHFR{NM_001290354.2}:r. 1_813_MSH3{NM_002439.	13
		5}:r.1417_4443	
80.633.876	80.656.410	DHFR{NM_001290354.2}:r. 1_929_MSH3{NM_002439.	8
		5}:r.314_4443	
80.649.388	80.656.410	DHFR{NM_001290354.2}:r. 1_686_MSH3{NM_002439.	6
		5}:r.314_4443	
80.649.388	80.678.926	DHFR{NM_001290354.2}:r. 1_686_MSH3{NM_002439.	2
		5}:r.1250_4443	
80.633.876	80.767.932	DHFR{NM_001290354.2}:r. 1_929_MSH3{NM_002439.	2
		5}:r.1973_4443	
80.649.388	80.787.564	DHFR{NM_001290354.2}:r. 1_686_MSH3{NM_002439.	1
		5}:r.2512_4443	
80.649.388	80.854.129	DHFR{NM_001290354.2}:r. 1_686_MSH3{NM_002439.	1
		5}:r.2890_4443	

The intrachromosomal fusion transcript comprised exons 1 to 4 of DHFR, which were juxtaposed to exon 9 of MSH3 (Figure 8B). Interestingly, the same fusion transcript, with a slightly different position, was already detectable at a lower extent in the U-2OS/MTX100 variant, suggesting that it was selected due to its involvement in MTX unresponsiveness. (Supplementary Table S4).

## 4 Discussion

In the present study, the pharmacogenomic profiling of MTX-resistant and -sensitive human HGOS cell lines has been performed by mmNGS targeting 22 SNPs with reported evidence to be related to the MTX-based chemotherapy response or toxicity development by mainly pharmacogenetic studies in HGOS patients. This innovative approach has provided novel biological insights and confirmed previously reported findings.

The validation of the custom mmNGS panel for variant calling by TaqMan genotyping assays was satisfactory, confirming the reliability of this methodology. Interestingly, the genotype distribution of the 22 SNPs was very heterogeneous, particularly being a homozygous wild type for all transporter genes except ABCB1. Different from the transporter genes, MTHFR rs1801133 and TP53 rs1042522 were most frequently found with variant alleles.

Germline and somatic TP53 mutations have been studied in patients with HGOS by several authors (Gokgoz et al., 2001; Pakos et al., 2004; Savage et al., 2007; Toffoli et al., 2009). HGOS often occurs in patients with Li–Fraumeni syndrome, a syndrome that leads to a predisposition to certain rare tumors, in which TP53 mutations are identified in 70% of patients (Savage et al., 2007; Mirabello et al., 2015). TP53 plays crucial roles in cell growth, apoptosis, and DNA repair, and mutations that occur in this gene could cause a loss of the protein tumor suppressor, leading to a more aggressive tumor. In patients with HGOS, TP53 has been reported to be somatically mutated in many cases (Gokgoz et al., 2001), which could be associated with reduced survival in osteosarcoma patients (Pakos et al., 2004).

The data obtained in the present study have confirmed the high instability of TP53 in HGOS cell drug-sensitive cell lines and MTX-resistant variants. However, the revealed TP53 changes could not directly be attributed to the development of MTX resistance.

One study reported a significant correlation between TP53 mutations and increased levels of DHFR mRNA in patients with acute lymphoblastic leukemia (ALL) (Goker et al., 1995), suggesting that the amplification of DHFR and TP53 mutations might be correlated with MTX resistance in ALL patients.

Among the 22 SNPs investigated in this study, MTHFR rs1801133 was found to be a variant in all eight MTX-resistant cell lines. This SNP caused an amino acid change which was reported to increase the thermolability of MTHFR protein, with a consequent decreased enzyme activity, leading to elevated plasma homocysteine levels and altered distribution of folate (Hider et al., 2007). In addition, MTHFR rs1801133 was also reported to be associated with liver toxicity in patients with HGOS due to the slower folate metabolism (Hattinger et al., 2020). MTHFR is an enzyme that catalyzes the conversion of 5,10-methylene-tetrahydrofolate to 5-methyl-tetrahydrofolate. The MTHFR rs1801133 Ala-Val mutation was reported to cause a decrease in the binding affinity of the MTHFR enzyme to the flavin adenine dinucleotide (FAD) cofactor by protein modeling and molecular dynamics studies (Abhinand et al., 2016). This amino acid change did not act directly on the kinetic properties of MTHFR but modified the ability of the MTHFR enzyme to bind FAD, causing a lower capacity for the remethylation of homocysteine into methionine. The mutation caused hyperhomocysteinemia with an increased cardiovascular risk and impairment of DNA methylation, with a consequent alteration of gene expression. In our study, the change of MTHFR rs1801133 to its homozygous wild-type observed in the two U-2OS variants with the higher levels of MTX resistance suggested that functional MTHFR is necessary for MTX resistance.

The reduced expression of SLC19A1 was reported as a mechanism underlying MTX resistance in MTX-resistant HGOS cell lines (Serra et al., 2004). These data have been confirmed by the mmNGS approach in the present study.

In addition, the sequence alterations of the entire coding region of the SLC19A1 gene were studied in 162 patients with different types of osteosarcoma (Yang et al., 2003). Interestingly, the authors defined the heterozygous status of rs1051266 SNP (A/G) as the wild type, according to the CCRF-CEM cell line, which transported MTX normally. This genotype was found in 37.6% (n = 61) of patients, followed by the homozygous GG status in 30.2% (*n* = 49) and the homozygous AA genotype in 22.8%. According to this definition in relation to the MTX transport, the detected change of rs1051266 to TT on DNA and RNA levels observed in all four Saos-2-derived MTX-resistant variants could, therefore, be associated with the altered MTX transport. However, it must be underlined that Yang et al. (2003) did not find an association between rs1051266 SNP and different responses to pre-operative chemotherapy, including MTX.

In other cancers, many fusion transcripts have been reported in association with drug resistance (Panicker et al., 2021), and it has been suggested that these fusion genes could be excellent targets to reestablish the sensitivity to chemotherapeutic drugs. In the present study, we have revealed a fusion transcript involving DHFR, a gene known to be responsible for MTX resistance, and MSH3, so far not known as drug resistance associated gene. This is, to the best of our knowledge, the first time that this fusion transcript has been reported in HGOS. The fact that we have identified this DHFR-MSH3 fusion transcript in the two U-2OS variants with the highest MTX resistance levels indicated an important role in the development of MTX resistance. Whether this DHFR-MSH3 fusion could also occur in HGOS tumors treated with high doses of MTX and not only in MTX-resistant cell lines remains to be elucidated.

So far, two studies have reported that in MTX-resistant cell lines, the promyelocytic leukemia HL-60 cell line (Drummond et al., 1997) and the colon cancer cell line HT-29 (Kim et al., 2021), DHFR and MSH3 were often co-amplified, leading to an overexpression of both genes. DHFR overexpression was described to elude the metabolic block caused by MTX, while the increased expression of MSH3 could significantly alter the concentration of MuTSα and MutSβ by decreasing the efficiency of the mismatch repair (Marra et al., 1998). In addition, in MTX-resistant HT-29 cells, Kim et al. (2021) identified several novel mutations and structural variants like duplications, deletions, and inversions in this site.

Fusion genes and RNAs are a hallmark of cancer and are the result from either chromosomal rearrangements or splicing mechanisms (Taniue and Akimitsu, 2021). The functional role of the fusion transcripts of adjacent genes is, however, still unclear, even though thousands of them have been deposited in several databases. A fusion transcript of DHFR and MSH3 has not been described in the literature to date but its presence in the two cell lines, with the highest levels of MTX resistance, suggests that it is a concomitant event together with the over-expression and amplification of DHFR previously identified by traditional methods (Hattinger et al., 2009).

Shoshani and co-workers recently showed how selective the pressure of MTX in HeLa S3 cells stimulated several rounds of chromothripsis, leading to the amplification of DHFR (Shoshani et al., 2021). HGOS are tumors in which high-confidence chromothripsis has been confirmed in 77% of the samples analyzed by whole-genome sequencing (Cortes-Ciriano et al., 2020), further suggesting that chromothripsis could underlie the development of MTX resistance.

The overexpression of MSH3 was not investigated because no primers were included in the mmNGS panel. However, our unpublished expression array data suggested that MSH3 was 1.99-fold upregulated in MTX-resistant variants compared to the parental U-2OS cell line, although these findings need to be further confirmed.

In conclusion, although HGOS has been studied by whole-genome sequencing techniques, the customized mmNGS approach targeting 22 SNPs of 12 genes with pharmacogenomic evidence reliably identified allele changes by variant calling on DNA- and RNA-derived libraries, differentially expressed genes, and a novel fusion RNA in relation to MTX resistance development.

## Data Availability

The next-generation-sequencing, multimodal-targeted datasets presented in this article are not readily available because the custom multimodal targeted panel has been designed also for additional targets, which have not been included in the submitted manuscript and have also not yet been published. Justified requests to access the datasets should be directed to the corresponding author.

## Abbreviations

ABCB1, ATP-binding cassette subfamily B member 1; ABCC2, ATP-binding cassette subfamily C member 2; ALL, acute lymphoblastic leukemia; CLC GWB, CLC genomics workbench; DHFR, dihydrofolate reductase; DME, drug-metabolizing enzyme; FAD, flavin adenine dinucleotide; FISH, fluorescence *in situ* hybridization; GGH, gamma-glutamyl hydrolase; HGOS, high-grade osteosarcoma; IMDM, Iscove’s modified Dulbecco’s medium; mmNGS, multimodal-targeted next-generation sequencing; MSH3, MutS homolog 3; MTHFD1, methylenetetrahydrofolate dehydrogenase; MTHFR, methylenetetrahydrofolate reductase; MTX, methotrexate; PCA, principal component analysis; SLC19A1/RFC1, solute carrier family 19 member 1/replication factor C subunit 1; SLC22A7, solute carrier family 22 member 7; SLC22A17, solute carrier family 22 member 17; SLC28A3, solute carrier family 28 member 3; SLCO1B1, solute carrier organic anion transporter family member 1B1; SNPs, single-nucleotide polymorphisms; TP53, tumor protein p53; VAF, variant allele frequency.
