# Supplementary material for: Single-nucleotide polymorphism profiling by multimodal-targeted next-generation sequencing in methotrexate-resistant and -sensitive human osteosarcoma cell lines
Source: Front Pharmacol. 2023 Nov 22;14:1294873. doi: 10.3389/fphar.2023.1294873 (PMC10698553; doi:10.3389/fphar.2023.1294873)
Supplement: Supplementary file 1 [file DataSheet2.PDF]

TABLE S3 Variations of 22 polymorphisms identified by TaqMan genotyping and multimodal targeted next generation sequencing in 20 human osteosarcoma cell lines

Polymorphisms	U-2OS		Saos-2		U-2OS/MTX3		U-2OS/MTX30		U-2OS/MTX100		U-2OS/MTX300		Saos-2/MTX30		Saos-2/MTX100		Saos-2/MTX300		Saos-2/MTX1µg	
	TaqMan	NGS	TaqMan	NGS	TaqMan	NGS	TaqMan	NGS	TaqMan	NGS	TaqMan	NGS	TaqMan	NGS	TaqMan	NGS	TaqMan	NGS	TaqMan	NGS
ABCB1 rs1045642			GA	GA									GA	GA	GA	GA	GA	GA	GA	GA
ABCB1 rs2032582	CA	CA	CA	CA	CA	CA	CA	CA	CA	CA	CA	CA	CA	CA	CA	CA	CA	CA	CA	CA
ABCB1 rs1128503	GG	GG	GA	GA	GG	GG	GG	GG	GG	GG	GG	GG	GA	GA	GA	GA	GA	GA	GA	GA
ABCC2 rs717620																				
ABCC2 rs2273697																				
ABCC2 rs3740066	CT	CT	TT	TT	CT	CT	CT	CT	CT	CT	CT	CT	TT	TT	TT	TT	TT	TT	TT	TT
ABCC2 rs17222723																				
DHFR rs1650723	/		/		/		/		/		/		/		/		/		/	
GGH rs1800909																				
GGH rs11545078																				
MTHFD1 rs2236225			AA	AA									AA	AA	AA	AA	AA	AA	AA	AA
MTHFR rs1801131	GT	GT			GT	GT	GT	GT	GT	GT	GT	GT								
MTHFR rs1801133	GA	GA	AA	AA	GA	GA	GA	GA	GA	GA	GA	GA	AA	AA	AA	AA	AA	AA	AA	AA
SLC19A1 rs1051266	/		/	CT	/		/		/		/		/		/		/		/	
SLC22A7 rs4149178																				
SLC22A17 rs4982753	CT	CT			CT	CT	CT	CT	CT	CT	CT	CT								
SLC28A3 rs885004																				
SLC28A3 rs7853758	/		/		/		/		/		/		/		/		/		/	
SLCO1B1 rs4149056																				
SLCO1B1 rs11045879																				
TP53 rs1042522	CC	CC	no amp.	no amp	CC	CC	CC	CC	CC	CC	CC	CC	no amp.	no amp.	no amp.	no amp.	no amp.	no amp.	no amp.	no amp.
TP53 rs1642785	CC	CC	no amp.	no amp	CC	CG	CC		CC		CC	CG	no amp.	no amp.	no amp.	no amp.	no amp.	no amp.	no amp.	no amp.

Polymorphisms	IOR/OS9		IOR/OS10		IOR/OS14		IOR/OS15		IOR/OS18		IOR/OS20		IOR/MOS		IOR/SARG		HOS		MG-63	
	TaqMan	NGS	TaqMan	NGS	TaqMan	NGS	TaqMan	NGS	TaqMan	NGS	TaqMan	NGS	TaqMan	NGS	TaqMan	NGS	TaqMan	NGS	TaqMan	NGS
ABCB1 rs1045642	GA	GA	GA	GA	GG	GG	GG	GG	GA	GA			GG	GG	GG	GA	GG	GG		
ABCB1 rs2032582	CA	CA	CA	CA	CC	CC	CC	CC	CA	CA			CC	CC	CC	CC	CC	CC		
ABCB1 rs1128503	GA	GA	GA	GA	GG	GG	GG	GG	GA	GA			GG	GG	GG	GG	GG	GG		
ABCC2 rs717620			CT	CT									CT	CT					CT	CT
ABCC2 rs2273697			GA	GA	AA	AA					AA	AA			GA	GA				
ABCC2 rs3740066			TT	TT									CT	CT	CT	CT			CT	CT
ABCC2 rs17222723																				
DHFR rs1650723	/		/	CT	/		/		/		/		/		/		/	CT	/	
GGH rs1800909	GG	GG			AA	GA			GA	GA	GG	GG			GA	GA			GA	GA
GGH rs11545078	AA	AA													GA	GA				
MTHFD1 rs2236225	GA	GA	GA	GA			GA	GA			GA	GA			GA	GA				
MTHFR rs1801131			GT	GT			GT	GT			GT	GT					GG	GG	GT	GT
MTHFR rs1801133	AA	AA	GA	GA	AA	AA			AA	AA	GA	GA	AA	AA	AA	AA				
SLC19A1 rs1051266	/	CT	/	CC	/	CT	/	CC	/	CT	/	CT	/	CC	/	CC	/	CC	/	CT
SLC22A7 rs4149178	GG	GG	GA	GA	GG	GG	GA	GA	GA	GA	GA	GA					GA	GG		
SLC22A17 rs4982753					CT	CT	CT	CT			CT	CT	CT	CT	TT	TT			TT	TT
SLC28A3 rs885004																	AA	AA	AA	AA
SLC28A3 rs7853758	/		/		/		/		/		/		/		/		/	AA	/	AA
SLCO1B1 rs4149056					CC	CC					CT	CT							CT	CT
SLCO1B1 rs11045879					CC	CC					CT	CT							CT	CT
TP53 rs1042522					CC	CC			CC	CC	CC	CC	CC	CC	CC	CC	CC	CC		
TP53 rs1642785					CC	CC			no amp.	no amp.										

no. amp.: no amplification
