# Supplementary material for: Single-nucleotide polymorphism profiling by multimodal-targeted next-generation sequencing in methotrexate-resistant and -sensitive human osteosarcoma cell lines
Source: Front Pharmacol. 2023 Nov 22;14:1294873. doi: 10.3389/fphar.2023.1294873 (PMC10698553; doi:10.3389/fphar.2023.1294873)
Supplement: Supplementary file 2 [file DataSheet3.PDF]

TABLE S4 Report of the fusion transcript between DHFR and MSH3 in U-2OS/MTX100 cell line

Fusion name	DHFR-MSH3
5' gene	DHFR
5' chromosome	5
3' gene	MSH3
3' chromosome	5
Reported transcript 5'	NM_001290354.2
Reported transcript 3'	NM_002439.5
Translocation name	DHFR{NM_001290354.2}:r.1_686_MSH3{NM_002439.5}:r.314_4443
P-value	1,00
Z-score	-∞
Fusion crossing reads	3
5' read coverage	42470
3' read coverage	3
