# Supplementary material for: Single-nucleotide polymorphism profiling by multimodal-targeted next-generation sequencing in methotrexate-resistant and -sensitive human osteosarcoma cell lines
Source: Front Pharmacol. 2023 Nov 22;14:1294873. doi: 10.3389/fphar.2023.1294873 (PMC10698553; doi:10.3389/fphar.2023.1294873)
Supplement: Supplementary file 3 [file DataSheet1.PDF]

Table S1. List of the genes selected for this study and their functions.

Gene Name	Abbreviation	Function
ATP Binding Cassette Subfamily B Member 1	ABCB1	Methotrexate transport
ATP Binding Cassette Subfamily C Member 2	ABCC2	Methotrexate transport
Dihydrofolate Reductase	DHFR	Folate metabolism
Gamma-Glutamyl Hidrolase	GGH	Folate metabolism
Methylenetetrahydrofolate Dehydrogenase	MTHFD1	Folate metabolism and hematological toxicity
Methylenetetrahydrofolate Reductase	MTHFR	Folate metabolism and liver toxicity
Solute Carrier Family 22 Member 7	SLC22A7	Methotrexate transport
Solute Carrier Family 22 Member 17	SLC22A17	Transport
Solute Carrier Family 19 Member 1/Replication Factor C subunit 1	SLC19A1/RFC1	Methotrexate transport
Solute Carrier Family 28 Member 3	SLC28A3	Pyrimidine and purine transport
Solute Carrier Organic Anion Trasporter Family Member 1B1	SLCO1B1	Methotrexate transport
Tumor Protein 53	TP53	Genomic stability

TABLE S2 List of TaqMan assays used for the genotyping analysis and reference alleles for variant identification by multimodal targeted next generation sequencing (mmNGS)

Gene_reference number	Assay ID	Type	Reference
ABCB1_rs1045642	C___7586657_20	DME	A
ABCB1_rs2032582	C_11711720C_30 + C_11711720D_40	DME	A
ABCB1_rs1128503	C___7586662_10	DME	A
ABCC2_rs717620	C___2814642_10	DME	C
ABCC2_rs2273697	C__22272980_20	DME	G
ABCC2_rs3740066	C__11214910_20	DME	C
ABCC2_rs17222723	C__25591743_30	DME	T
DHFR rs1650723	Not done	Not done	C
GGH_rs1800909	C___8894713_10	Funct. tested	A
GGH_rs11545078	C__25623170_10	Funct. tested	G
MTHFD1_rs2236225	C___1376137_10	Funct. tested	G
MTHFR _rs1801131	C____850486_20	Funct. tested	T
MTHFR_rs1801133	C___1202883_20	Funct. tested	G
SLC19A1/RFC1_rs1051266	Not done	Not done	T
SLC22A7_rs4149178	C__11542962_10	Validated	A
SLC22A17_rs4982753	C___2484726_10	Validated	C
SLC28A3_rs885004	C___2752627_10	Validated	G
SLC28A3_rs7853758	Not done	Not done	G
SLCO1B1_rs4149056	C__30633906_10	DME	T
SLCO1B1_rs11045879	C__31106904_10	Funct. tested	T
TP53_rs1042522	C___2403545_10	Funct. tested	G
TP53_rs1642785	C___2880090_10	Funct. tested	G

DME: Drug-metabolizing enzyme, Funct. tested: Functionally tested
